# Safety and effectiveness of antimalarial therapy in sickle cell disease: a systematic review and network meta-analysis

**DOI:** 10.1186/s12879-018-3556-0

**Published:** 2018-12-12

**Authors:** Augustina Frimpong, Laty Gaye Thiam, Benjamin Arko-Boham, Ewurama Dedea Ampadu Owusu, George O. Adjei

**Affiliations:** 1 West African Centre for Cell Biology of Infectious Pathogens, Department of Biochemistry Cell and Molecular Biology, University of Ghana, Accra, Ghana; 2 Department of Immunology, Noguchi Memorial Institute for Medical Research, University of Ghana, Accra, Ghana; 3 African Institute for Mathematical Sciences, Cape Coast, Ghana; 4 Department of Medical Laboratory Sciences, School of Biomedical and Allied Health Sciences, College of Health Sciences, University of Ghana, Accra, Ghana; 5 Foundation for Innovative New Diagnostics (FIND), Geneva, Switzerland; 6 Centre for Tropical Clinical Pharmacology and Therapeutics, School of Medicine and Dentistry, University of Ghana, Accra, Ghana; 7 Office of Research Innovation and Development, University of Ghana, Accra, Ghana

**Keywords:** Sickle cell disease, Malaria, Chemoprophylaxis, Safety, Effectiveness, Adverse events

## Abstract

**Background:**

About 80% of all reported sickle cell disease (SCD) cases in children anually are recorded in Africa. Although malaria is considered a major cause of death in SCD children, there is limited data on the safety and effectiveness of the available antimalarial drugs used for prophylaxis. Also, previous systematic reviews have not provided quantitative measures of preventive effectiveness. The purpose of this research was to conduct a systematic review and meta-analysis of the available literature to determine the safety and effectiveness of antimalarial chemoprophylaxis used in SCD patients.

**Methods:**

We searched in PubMed, Medline, CINAHL, POPLine and Cochrane library, for the period spanning January 1990 to April 2018. We considered randomized or quasi-randomized controlled trials comparing any antimalarial chemoprophylaxis to, 1) other antimalarial chemoprophylaxis, 2) placebo or 3) no intervention, in SCD patients. Studies comparing at least two treatment arms, for a minimum duration of three months, with no restriction on the number of patients per arm were reviewed. The data were extracted and expressed as odds ratios. Direct pairwise comparisons were performed using fixed effect models and the heterogeneity assessed using the I-square.

**Results:**

Six qualified studies that highlighted the importance of antimalarial chemoprophylaxis in SCD children were identified. In total, seven different interventions (Chloroquine, Mefloquine, Mefloquine artesunate, Proguanil, Pyrimethamine, Sulfadoxine-pyrimethamine, Sulfadoxine-pyrimethamine amodiaquine) were evaluated in 912 children with SCD. Overall, the meta-analysis showed that antimalarial chemoprophylaxis provided protection against parasitemia and clinical malaria episodes in children with SCD. Nevertheless, the risk of hospitalization (OR = 0.72, 95% CI = 0.267–1.959; I^2^ = 0.0%), blood transfusion (OR = 0.83, 95% CI = 0.542–1.280; I^2^ = 29.733%), vaso-occlusive crisis (OR = 19, 95% CI = 1.713–2.792; I^2^ = 93.637%), and mortality (OR = 0.511, 95% CI = 0.189–1.384; I^2^ = 0.0%) did not differ between the intervention and placebo groups.

**Conclusion:**

The data shows that antimalarial prophylaxis reduces the incidence of clinical malaria in children with SCD. However, there was no difference between the occurrence of adverse events in children who received placebo and those who received prophylaxis. This creates an urgent need to assess the efficacy of new antimalarial drug regimens as potential prophylactic agents in SCD patients.

**Systematic review registration:**

PROSPERO (CRD42016052514).

**Electronic supplementary material:**

The online version of this article (10.1186/s12879-018-3556-0) contains supplementary material, which is available to authorized users.

## Introduction

Sickle cell disease (SCD) is a group of autosomal recessive inherited disorders caused by a single nucleotide substitution (T > A) in the β-globin gene. The mutation (Glu6Val) promotes polymerization of deoxygenated sickle haemoglobin S (HbS) and decreased deformability of red blood cells resulting in obstruction of the vasculature and a range of pathophysiological effects including vaso-occlusion, organ ischaemia, infarction, and early mortality [1]. SCD accounts for 5–19% mortality in children in sub-Saharan Africa, where the highest frequency of homozygous SCD occurs [2]. SCD-related mortality occurs mostly in undiagnosed infants in sub-Saharan Africa, and is predominantly attributed to infections, including malaria [3]. Although a linkage exists between the presence of sickle haemoglobin (HbS) and protection from malaria in the heterozygous state [4], malaria is a frequent cause of hospitalization and poor outcome among children with SCD in endemic areas [5, 6], and malaria is associated with a higher mortality in hospitalized SCD patients compared to hospitalized non-SCD patients [7–9]. Prophylaxis against malaria is therefore important in SCD patients, as antimalarial chemoprophylaxis has also been shown to be beneficial in SCD patients, reducing parasitaemia and anaemia, and the requirement for blood transfusion [10–12].

The WHO recommends that SCD patients in endemic areas should receive antimalarial prophylaxis [13, 14]; however, the evidence to support the potential beneficial effects of this strategy in SCD patients is limited: i) almost all previous individual studies were underpowered to detect some clinically important outcomes, and ii) whereas an early systematic review included two small studies [15], a latter review that included six randomized trials provided descriptive information [16]. There is no consensus on the optimal chemoprophylactic regimen for SCD patients especially in the context where artemisinin-based regimens are the treatment standard. There is also lack of data on the comparative effect of different antimalarial chemoprophylactic regimens that have never been compared directly in randomized clinical trials, and data on the overall effect of chemoprophylactic regimens in trials with more than two arms are non-existent. In the absence of direct head-to-head comparisons on interventions, network meta-analysis provides a statistical framework that incorporates evidence from both direct and indirect comparisons from a network of studies of different therapies to evaluate their relative effects. This systematic review was conducted to provide an update on existing evidence based on new studies published and compare drugs that have been used in this context.

## Methods

### Search strategy and selection criteria

We considered randomized or quasi-randomized controlled trials comparing any antimalarial chemoprophylactics to, 1) other antimalarial chemoprophylactics, 2) placebo or 3) no intervention, in SCD patients. The selection included studies that compared at least two treatment arms, for a minimum duration of three months, with no restriction on the number of patients per arm. The search was conducted between May 2016 and April 2018 in, PubMed, Medline, CINAHL, POPLine and Cochrane library, for the period spanning January 1990 to April 2018. The MeSH terms for the search included a combination of the following terms, *sickle cell disease, malaria, malaria chemoprophylaxis, chemoprophylactics, malaria chemotherapy(y)ies, mortality, hospitalization, morbidity, effectiveness of, antimalarial therapy, antimalarial drugs, malaria therapy, malaria treatment, malaria prophylaxis, safety of, safety and effectiveness, randomized trials, randomized control trials, quasi-randomized trials*. Studies that met the inclusion criteria and published in English were included. Non-peer reviewed articles and other publication types were used to guide in the search for further literature (technical reports, theses, working papers). We also contacted authors for supplementary information relating to the haematological parameters. Citations and articles were downloaded and organized using EndNote (versionX5).

### Data screening and study selection

Two independent reviewers conducted the search, screened titles and abstracts and removed duplicate publications. However, no informative result was obtained from the grey literature search. Studies that did not indicate the use of any of the interventions were excluded. Subsequently, full-text articles were retrieved, screened and retained if they met the inclusion criteria. The two independent reviewers extracted data with data abstraction forms which included details about the study design, sample size, recruitment method, data characteristics, location, study period, interventions, and outcome. All disagreements on study inclusion criteria were referred to a third member of the study team for resolution.

### Data extraction and quality assessment

The quality of the studies was assessed using the Cochrane Risk of Bias tool, identifying the presence or otherwise of key concepts or themes relating to antimalarial intervention trials including, *random sequence generation, allocation concealment, blinding of participants, blinding of outcome assessment, incomplete data reporting, selective reporting, and other bias* [17].

### Outcome measures

The pre-specified primary outcome measures were, 1) safety and 2) effectiveness of the antimalarial chemoprophylactics for SCD patients. Safety was defined as, the ability of the intervention to minimize the occurrence of undesirable outcomes (adverse events, changes in haematological parameters) and, effectiveness, as the ability of the intervention a) to decrease detectable parasitaemia and/or b) to limit the number of clinical malaria episodes. Other outcome measures included mortality as associated with a clinical malaria episode, or vaso-occlusive crisis (VOC), and the number of VOC episodes.

### Statistical analysis

The Comprehensive meta-analysis software version 3 was used to conduct direct pair-wise meta-analyses using placebo as a control. Outcomes were expressed as odds ratios and fixed effect methods were used to estimate the mean and 95% confidence intervals (CI). Heterogeneity was assessed using the I-squared (I^2^), and a network of randomized controlled trials was also conducted based on the frequentist approach using the netmeta package in R studio version 2 [18]. We performed direct and indirect pairwise comparisons which provided the odds ratio from a fixed effect model with 95% confidence intervals from the estimates. Treatments were ranked using the P-scores for the effectiveness of the intervention.

## Results

**Fig. 1:**
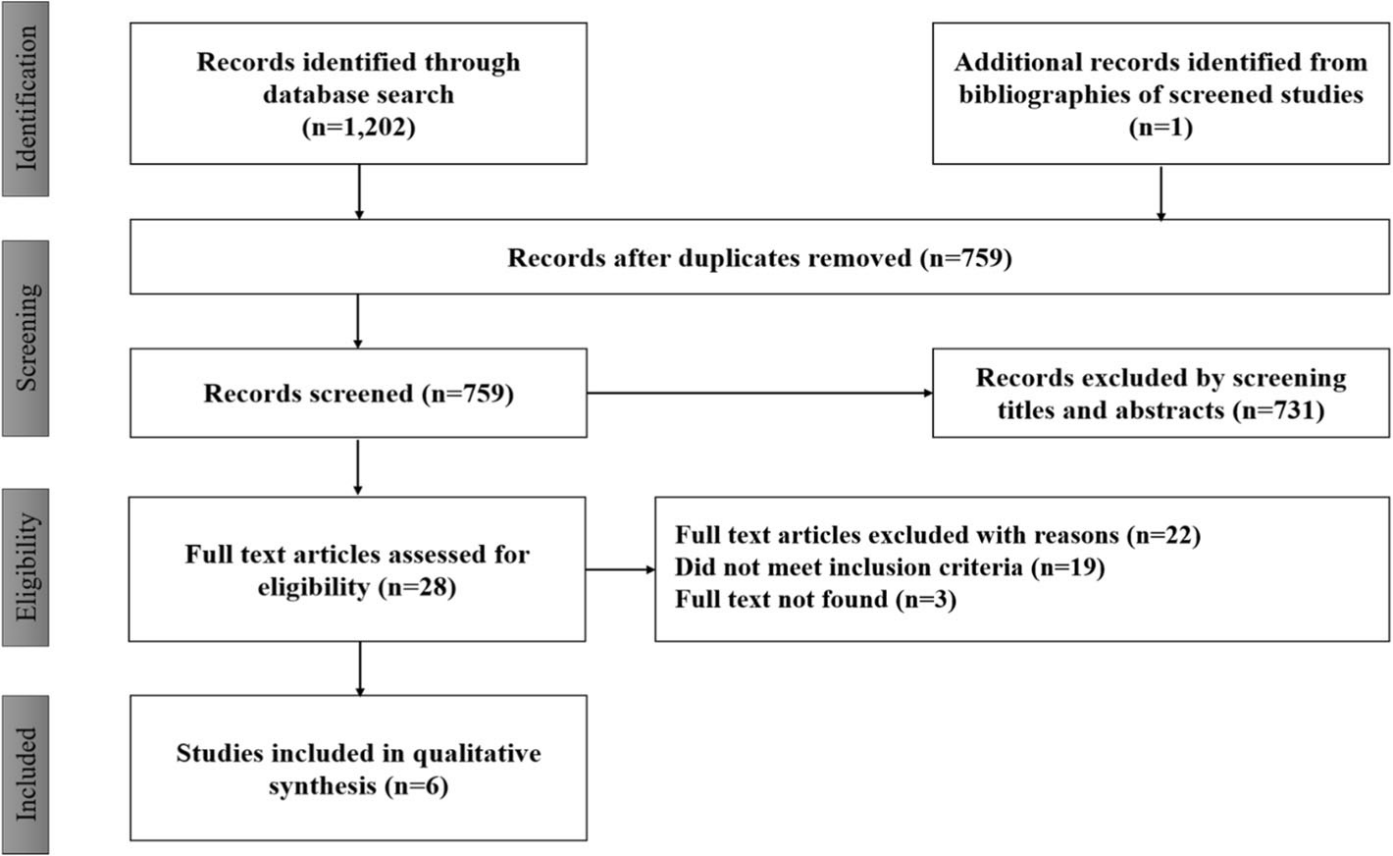
PRISMA flow chart for the systematic review of antimalarial drugs used for preventing malaria in sickle cell disease (SCD) patients

The initial search resulted in a total of 1202 records, of which 759 were retained after excluding duplicate publications. A total of five studies met the criteria for inclusion, and subsequently, an additional study (*n* = 1) was included from the bibliography of screened studies. The flow diagram for selection and inclusion of studies is shown (Fig. 1). A total of six randomized controlled trials, including two trials that included three arms [14, 19], and four trials that included two arms [20–23] were included in the meta-analysis. A total of 912 SCD patients were included in the six studies, and the following seven chemoprophylactic regimens [(a) Proguanil (PG); (b) Sulphadoxine-Pyrimethamine (SP); (c) Mefloquine (MQ); (d) Chloroquine (CQ); (e) Pyrimethamine (PM); (f) Mefloquine-Artesunate (MQAS); and (g) Sulphadoxine-Pyrimethamine-Amodiaquine (SPAQ)] were evaluated in the meta-analysis. A total of 831 enrolled participants completed follow up periods ranging from five months [21] to twenty-one months [23]. The largest number of participants in a trial were enrolled in trials that included PG (*n* = 182) and CQ (n = 182) arms, followed by trials that included an SP arm (*n* = 150), MQAS arm (*n* = 90), SPAQ arm (*n* = 90), MQ arm (*n* = 56), and PM (*n* = 36) (Table 1). The methodological quality of all included trials was assessed as good, allocation concealment was appropriate, and all but one study provided an adequate report on adverse events occurring in the respective treatment arms. However, only one of the studies [21] described an adequate blinding procedure (Table 2).

**Table 1:** Characteristics of studies included in this systematic review and meta-analysis

Author	Country	Study period (months)	Drug regimen	No. of participants	Genotype	Adverse events (%)	Patients with detectable parasitaemia and/or malaria clinical episodes (%)	No. of deaths	Success rate (95% CI)
Olaosebikan et al., 2015	Nigeria	14	MQAS	90	SS/SC	24	7.78	4	61% (compared to PG)
			SPAQ	90		13.8	13.33	0	36% (compared to PG)
			PG	90		5.4	21.11	3	–
Diop et al., 2010	Senegal	6	PL	30	SS	3.33	13.33	0	86.67%
			SP	30		3.33	0	0	100%
Nakibuuka et al., 2009	Uganda	5	SP	120	SS	6.6 and 1.6 (vomiting, Pruritus)	14	0	50% (compared to CQ)
			CQ	122		11.5 and 1.8 (vomiting, Pruritus)	24.6	0	–
Eke et al., 2003	Nigeria	9	PL	30	SS	Not reported	31	1	69%
			PM	36			38.9	0	61%
			PG	35			15.6	0	84%
Nwokolo et al., 2001	Nigeria	6	PG	57	SS	19.6	18.2	None	81.80%
			MQ	56		31.6	10.8	None	89.20%
Warley et al., 1965	Uganda	21	PL	66	SS	NA	31.82	None	68.18
			CQ + benzathine penicillin	60		NA	11.67	None	88.33

CQ = Chloroquine, MQ = Mefloquine, MQAS = Mefloquine-artesunate, NA = Not available, PL = Placebo, PG = Proguanil, PM = Pyrimethamine, SP=Sulfadoxine-pyrimethamine, SPAQ = Sulfadoxine pyrimethamine-amodiaquine, SS = homozygous sickle haemoglobin

**Table 2:** Assessment of risk of bias of studies included in the meta-analysis

Author details	Study site	Drug regimen	Allocation sequence	Allocation concealment	Blinding	No evidence of incomplete outcome data recording	No evidence of selective outcome recording
Olaosebikan et al., 2015	Kwara state, Nigeria	MQAS SPAQ PG	Yes	Yes	(Open-label) Laboratory staff were unaware of treatment allocation	MQAS (2 withdrew consent, 3 moved away, 12 lost and 4 deaths) SPAQ (1 withdrew consent, 2 moved away and 12 lost) PG (1 moved away, 26 lost and 3 deaths)	Yes ^b^
Diop et al., 2010	Dakar, Senegal	PL SP	Yes	Yes	Open-label	Yes^a^	Yes ^b^
Nakibuuka et al., 2009	Kampala, Uganda	SP CQ	Yes	Yes	Double-blind	SP (7 lost to follow up) CQ (8 lost to follow up)	Yes ^b^
Eke et al., 2003	Port Harcourt, Nigeria	PL PM PG	Yes	Yes	Open-label	Yes^a^	Yes ^b^
Nwokolo et al., 2001	Multicenter, Nigeria	PG MQ	Yes	Yes	Open-label	Yes^a^	Yes ^b^
Warley et al., 1965	Kampala, Uganda	PL CQ + benzathine penicillin	Yes	Yes	Not provided	Yes^a^	No (dactylitis and Haemoglobin)

CQ = Chloroquine, MQ = Mefloquine, MQAS = Mefloquine-artesunate, PL = Placebo, PG = Proguanil, SP=Sulfadoxine-pyrimethamine, SPAQ = Sulfadoxine pyrimethamine-amodiaquine

^a^Yes indicates that there is no evidence of incomplete outcome data recording

^b^ Yes indicates that there is no evidence of selective outcome recording

### Comparator drugs

**Fig. 2:**
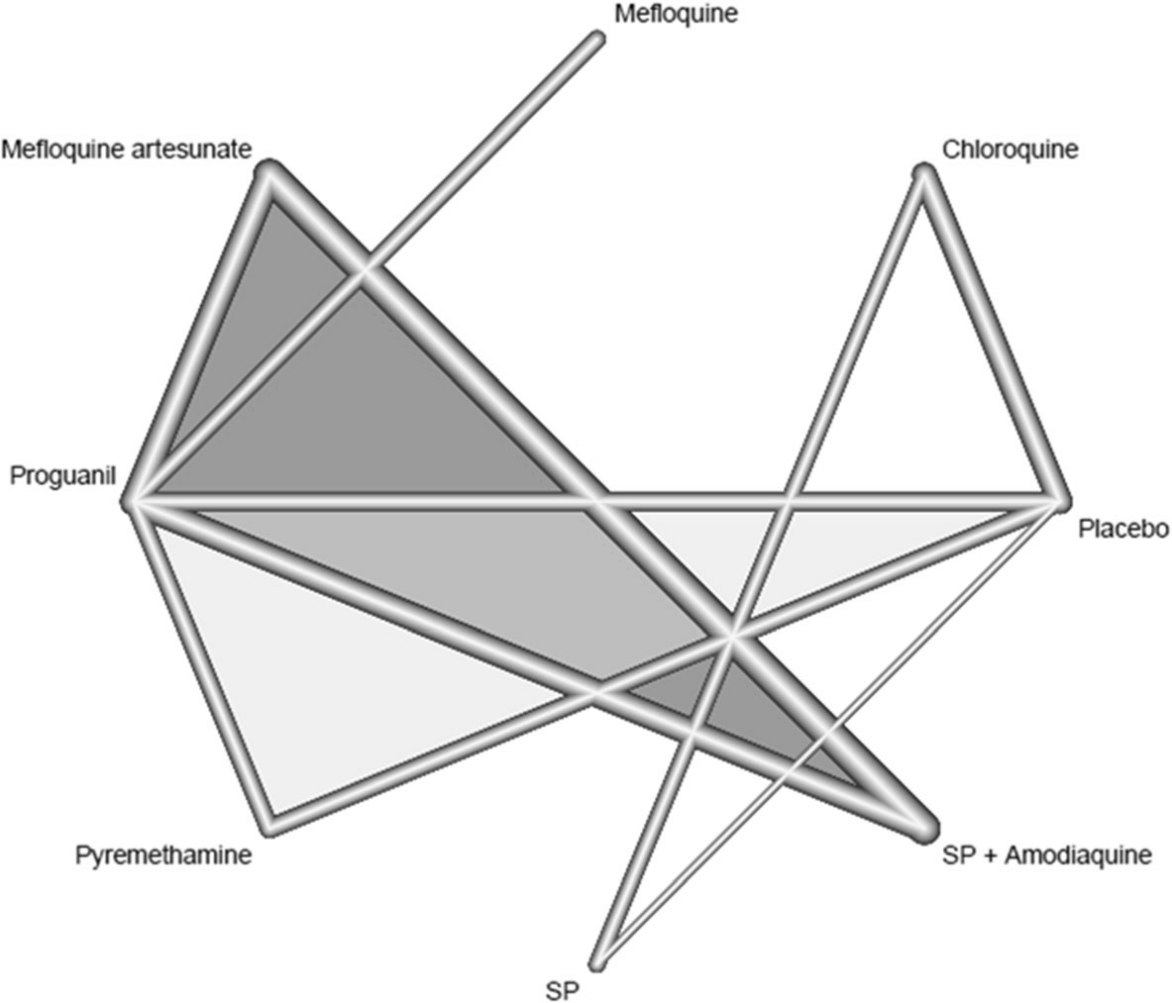
Network diagram of interventions included in the analysis. A network of eligible comparisons for the efficacy of the various treatments. Lines represent the presence of direct comparison trials. The width of the lines represent the number of participants included in the intervention groups

In all, PG was the most common active comparator drug and was also the most commonly evaluated stand-alone drug [14, 19, 22]: it was compared with four different interventions in three different studies (Table 1) [14, 19, 22]. SPAQ and MQAS were each compared with two different interventions in one single study [19], and CQ was evaluated as a stand-alone drug in one study [21], and in combination with benzathine penicillin in an early intervention study [23]. SP, MQ and PM, were each compared to a single intervention at a time in single studies (Table 1; Fig. 2). The network of eligible comparisons for outcome measures is shown (Fig. 2). Efficacy and tolerability were evaluated as outcome measures in six interventions out of the 21-possible pair-wise comparisons between the seven interventions. All the interventions (except studies evaluating MQAS, SPAQ or PG), included at least one placebo-controlled trial, and the majority of the interventions also directly compared at least one active comparator (Table 1).

### Parasitaemia and/or clinical malaria episodes

**Fig. 3:**
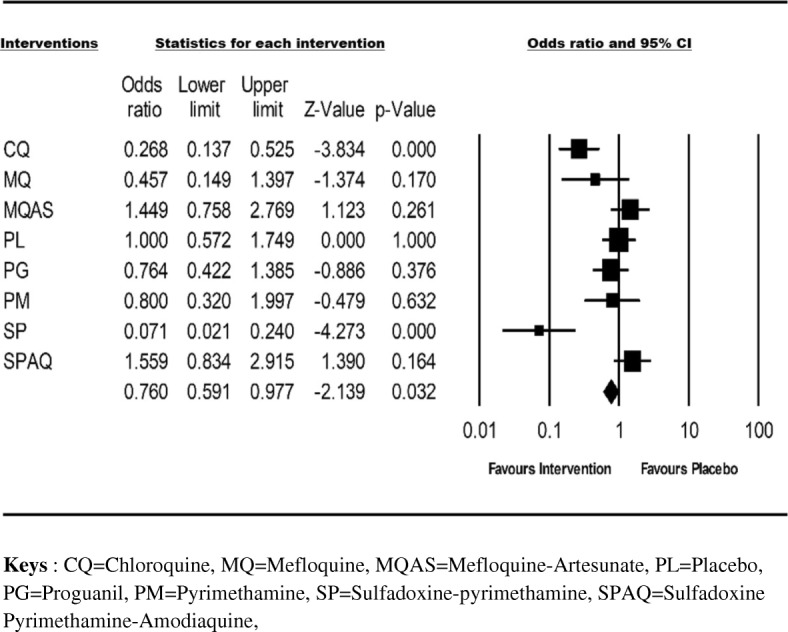
Summary plot showing the effectiveness of the chemoprophylaxis with placebo as a reference. Antimalarial drugs were compared to placebo with the risk of developing malaria. Each single drug was represented as a square. The square area denotes the contribution of the drug to the meta-analysis. Horizontal line denotes the odds ratio and the confidence interval. The diamond represents the combined odds ratio with confidence interval. Odds ratios lower than 1 favour interventions and 95% CI values (Lower limit and Upper limit) comprised between 0 and 1 is associated with a significant difference

All the included studies provided data on the number of clinical malaria episodes and/or the number of patients presenting with detectable parasitaemia during the follow-up period (Table 1). The cumulative odds ratio of the effectiveness of the interventions was 0.76 (95% CI = 0.591–0.97; I^2^ = 79.6%) (Fig. 3). Overall, SCD patients receiving prophylaxis were less likely to harbour detectable parasitaemia or to present with a clinical malaria episode compared to the placebo group (relative risk reduction of 24%) (Fig. 3). Taken individually, SP and CQ seemed to provide better protection compared to the rest of the interventions (Fig. 3 and Additional file 1: Figure S1).

### Adverse event occurrence

**Fig. 4:**
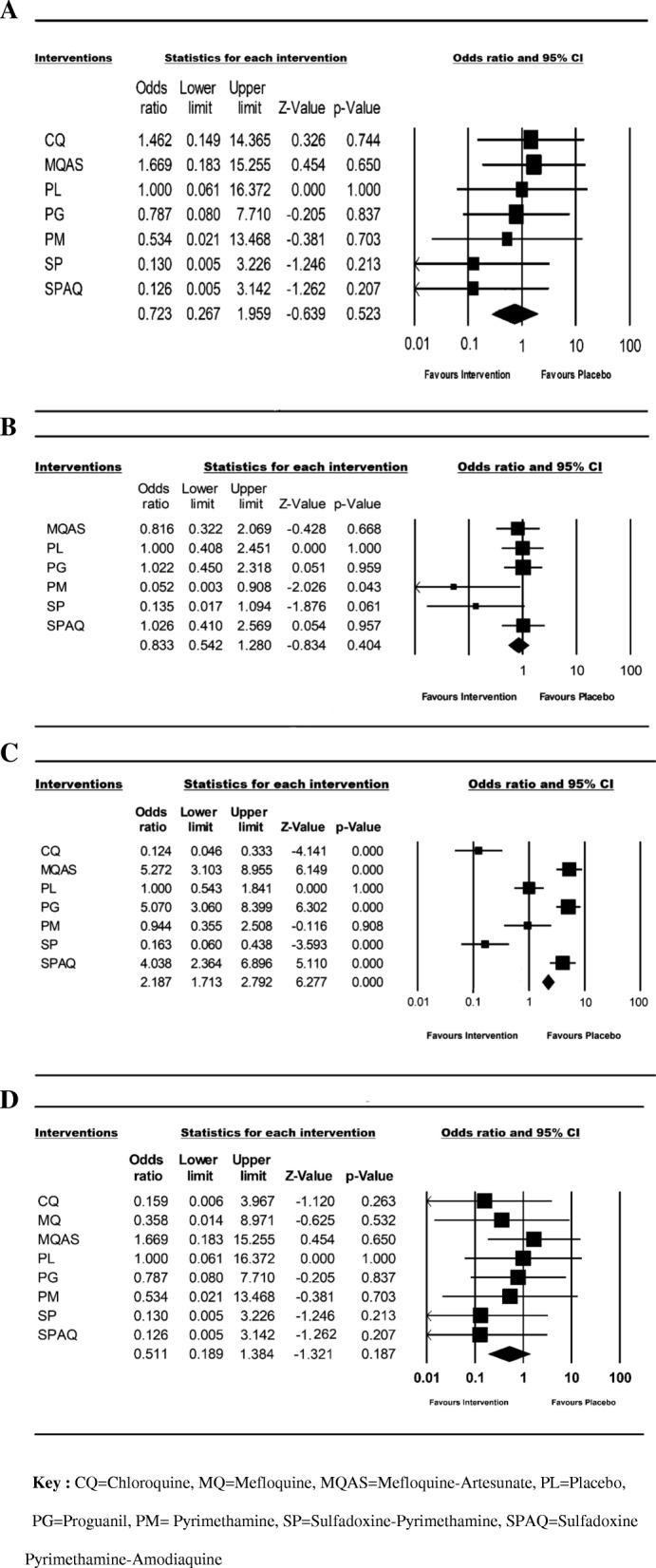
Forest plots showing the commonly reported adverse-events related to the antimalaria chemoprophylaxis in SCD patients. Results are shown for (**a**) hospitalization, (**b**) blood transfusion, (**c**) vaso-occlusive crisis and (**d**) mortality with placebo as a reference. The square area denotes the contribution of the drug to the meta-analysis. Horizontal line denotes the odds ratio together with the confidence intervals. The diamond represents the combined odds ratio with its confidence interval. Odd Ratios lower than 1 favour interventions and 95% CI values (Lower limit and Upper limit) comprised between 0 and 1 is associated with a significant difference

The majority of studies (a total of five out of six) provided information about adverse event occurrence (Table 1), Reported adverse events were mostly minor (e.g., vomiting, body pain, weakness, pruritus, headache, and nausea), with the most commonly reported major adverse events being, hospitalization, blood transfusion, vaso-occlusive crisis and mortality. The cumulative odds ratio of adverse event occurrence in the intervention arm was 0.72 (95% CI = 0.267–1.959; I^2^ = 0.0%) for hospitalisation (Fig. 4a); 0.83 (95% CI = 0.542–1.280; I^2^ = 29.733%) for blood transfusion (Fig. 4b); 2.19 (95% CI = 1.713–2.792; I^2^ = 93.637%) for vaso-occlusive crisis (Fig. 4c) and 0.511 (95% CI = 0.189–1.384; I^2^ = 0.0%) for mortality (Fig. 4d). Compared to placebo, all interventions were associated with a marginally reduced risk of hospitalization, except CQ and MQAS (95% CI for the population included 1.0 for all treatments) (Fig. 4a), and no significant difference in the risk of blood transfusion was present between those who received an intervention or placebo, except for PM recipients (Fig. 4b). Unexpectedly, the risk of vaso-occlusive crisis occurrence was more likely in PG, MQAS and SPAQ recipients compared to placebo, but less likely in those who received CQ and SP (Fig. 4c). All the studies reported mortality outcomes (Table 1). However, aside from the placebo group, mortality was reported in only two groups, (PG and MQAS), the latter reporting a higher mortality risk rate (Fig. 4d).

### Sickle cell crises and changes in hematological parameters

None of the eligible studies reported significant differences in the number of sickle cell crises or changes in hematological parameters (Additional file 2: Table S1).

## Discussion

This systematic review has provided an opportunity to provide quantitative data on the safety and efficacy of antimalarial prophylactic drugs in SCD patients, thus extending the available summary evidence on this indication. The network meta-analysis component has also enabled a comparison of all active comparator drugs used in clinical trials of antimalarial prophylaxis in SCD to date, thus, increasing the chances of generating an internally consistent set of estimates while respecting the randomization in the original trials. Due to the lack of standard basis to normalize the data from different studies while conducting the network meta-analysis, our main focus remained on the pairwise meta-analysis to assess both the efficacy and safety of individual interventions.

The results from this meta-analysis shows that anti-malarial chemoprophylaxis provided protection against parasitemia and clinical malaria episodes in children with SCD. However, the results show that the intervention did not have an effect on the risk of hospitalization, need for blood transfusion, vaso-occlusive crises and mortality in SCD patients.

In this study, we observed that PG was the commonest chemoprophylaxis used in three of the six studies [14, 19, 22], partly because these studies were conducted in Nigeria where PG is the recommended IPT for SCD patients. In addition, it could be due to the relative safety of this antimalarial drug [24], an important characteristic in such a vulnerable target group and for such an indication. However, parasitaemia was reported in all the studies in which PG was used as prophylaxis. This may be due to sub-optimal compliance, as PG is administered daily due to its relatively short half-life [25–27].

Although the results of the meta-analysis did not show a decrease in the incidence of blood transfusion and vaso-occlusive crises; an analysis of individual drugs showed a decreased in blood transfusion or anaemia in PM recipients. Also, the incidence of VOC was lower in CQ and SP recipients. The high likelihood of VOC observed in SCD patients on prophylaxis may be due to presence of *Plasmodium* infection or parasitemia. Despite the fact that the development of VOC requires the interplay of various factors [28], it may be exacerbated by malaria infection. However most of these studies were conducted in the era preceding the evolution of widespread resistance to these antimalarial drugs.

Due to this, the use of anti-malarial monotherapies as prophylaxis in a group as vulnerable as sickle cell disease patients is of utmost concern [29–32], as the two drugs (CQ and SP) that showed protective efficacy are no longer recommended for treatment because of widespread resistance. Although the use of combination therapy is currently recommended, available evidence indicates that the efficacy of such drugs (e.g., SPAQ and MQAS), were relatively low in SCD patients despite evidence of their increased efficacy in individuals with normal HbAA genotypes [33, 34]. Therefore, it is imperative that new studies with currently available antimalarial prophylactic drugs be conducted in SCD patients.

Also, the meta-analysis data showed a higher MQ-related adverse event incidence, both in studies in which MQ was evaluated as a stand-alone drug [22], as well as in studies in which the drug was used in combination with AS [19]. This finding is consistent with the dose-dependent association of MQ with neuropsychiatric adverse events [35]. Nevertheless, only one of the studies in which MQ was evaluated [19] provided details about the MQ dosing regimen. This finding may suggest that alternative prophylactic regimens other than mefloquine may be more desirable for prophylaxis in SCD patients.

We could not identify any previous studies evaluating the safety or efficacy of currently recommended ACT regimens as components of prophylactic regimens in SCD patients. The only available (two) studies [36, 37] that have reported on the safety of ACTs among children with haemoglobinopathies reported an improved outcome in some selected haematological indices following treatment with ACTs in these groups of children. However, as concluded by a recent systematic review on ACTs and malaria in haemoglobinopathies, the efficacy of these ACTs when used as prophylaxis in haemoglobinopathies is yet to be proven [38]. This implies that, there is still limited evidence on the safety and efficacy of antimalarial prophylaxis in SCD patients and therefore, highly recommended that new antimalarial drugs should be evaluated for their efficacy and safety specifically in SCD patients.

The limitations of this study include the fact that the analysis included 912 patients; while this sample size is modest and likely to affect the outcome of low-incidence variables (e.g., mortality), it is unlikely to affect outcomes such as parasitaemia or VOC. Furthermore, the limited number of studies, coupled with the small sample size of individual studies, as well as study quality and other implementation challenges could account for the observed substantial heterogeneity in the outcome parameters. These may have also accounted for the lack of association between safety parameter outcomes and antimalarial prophylaxis observed in the analysis.

## Conclusion

The data shows that the use of antimalarial drugs as prophylaxis in sickle cell disease patients results in reduction in incidence of malaria; whereas the incidence of hospitalisation, blood transfusion, vaso-occlusive crises and mortality were not different between SCD patients on prophylaxis compared to those on placebo. Nonetheless, using stand-alone drugs, SCD patients using PM, had a reduction in hospitalization compared to patients on any of the other prophylaxis or placebo, while CQ or SP prophylaxis were also associated with reduced occurrence of vaso-occlusive crises.

The findings indicate that there is non-existent data on currently recommended antimalarial drugs as prophylaxis in SCD patients thus, there is a need to evaluate the safety and efficacy of new antimalarial drugs including the utility of currently recommended ACT regimens, as potential prophylactic agents in SCD patients.

## Additional files

Additional file 1: **Figure S1.** Ranking the effectiveness of the interventions. A histogram plot showing the ranking probability on the effectiveness of the treatment. This was performed by using the point estimates and standard errors. SP=Sulfadoxine-Pyrimethamine, CQ = Chloroquine, MQ = Mefloquine, PG = Proguanil, PM = Pyrimethamine, PL = Placebo, MQAS = Mefloquine-Artesunate, SPAQ = Sulfadoxine Pyrimethamine-Amodiaquine. (DOCX 28 kb)

Additional file 2: **Table S1.** Haematological parameters for safety profile. (DOCX 17 kb)

## Abbreviations

ACT: Artemisinin combination therapy
CQ: Chloroquine
HbS: Haemoglobin S
IPT: Intermittent preventive threatment
MQ: Mefloquine
MQAS: Mefloquine-Artesunate
PM: Pyrimethamine
PQ: Proguanil
PRISMA: Preferred Reporting Items for Systematic Reviews and Meta-Analysis
SCD: Sickle cell disease
SP: Sulphadoxine-Pyrimethamine
SPAQ: Sulphadoxine-Pyrimethamine-Amodiaquine
VOC: Vaso-occlusive crisis
