# Supplementary material for: Safety and effectiveness of antimalarial therapy in sickle cell disease: a systematic review and network meta-analysis
Source: BMC Infect Dis. 2018 Dec 12;18:650. doi: 10.1186/s12879-018-3556-0 (PMC6292161; doi:10.1186/s12879-018-3556-0)
Supplement: Supplementary file 1 — Figure S1. Ranking the effectiveness of the interventions. A histogram plot showing the ranking probability on the effectiveness of the treatment. This was performed by using the point estimates and standard errors. SP=Sulfadoxine-Pyrimethamine, CQ = Chloroquine, MQ = Mefloquine, PG = Proguanil, PM = Pyrimethamine, PL = Placebo, MQAS = Mefloquine-Artesunate, SPAQ = Sulfadoxine Pyrimethamine-Amodiaquine. (DOCX 28 kb) [file 12879_2018_3556_MOESM1_ESM.docx]

**Figure S1: Ranking the effectiveness of the interventions.** A histogram plot showing the ranking probability on the effectiveness of the treatment. This was performed by using the point estimates and standard errors. SP=Sulfadoxine-Pyrimethamine, CQ=Chloroquine, MQ=Mefloquine, PG=Proguanil, PM= Pyrimethamine, PL=Placebo, MQAS=Mefloquine-Artesunate, SPAQ=Sulfadoxine Pyrimethamine-Amodiaquine
