# Supplementary material for: Safety and effectiveness of antimalarial therapy in sickle cell disease: a systematic review and network meta-analysis
Source: BMC Infect Dis. 2018 Dec 12;18:650. doi: 10.1186/s12879-018-3556-0 (PMC6292161; doi:10.1186/s12879-018-3556-0)
Supplement: Supplementary file 2 — Table S1. Haematological parameters for safety profile. (DOCX 17 kb) [file 12879_2018_3556_MOESM2_ESM.docx]

**Table S1: Haematological parameters for safety profile**

|  | Study site | Study span | Drug regimen | Control | No. of children | Mean Hb concentration | | Mean WBC count | | Mean platelet count |
| --- | --- | --- | --- | --- | --- | --- | --- | --- | --- | --- |
|  |  |  |  |  |  | **Baseline** | **End-point** | **Baseline** | **End-point** |  |
| Olaosebikan et al., 2015 | Kwara state, Nigeria | 14 months | MQAS  +  SPAQ | PG | 270 with 90 in each group | MQAS=7.4 SPAQ=7.5  PG=7.5 | MQAS=7.49 SPAQ=7.62 PG=7.76 | N/A | | N/A |
| Diop et al., 2010 | Dakar, Senegal | Sept, 2007-Feb, 2008 6 months | 3 ITP + SP OR 3ITP+Placebo | 3 ITP+PL | SCD=60 | 3 ITP+SP=7.9 ITP+PL=8.1 | Transfused units ITP+SP=1 ITP+PL=4 | SP=12.03  PL=11.8) | | SP=449.5 PLAC=453 |
| Warley et al., 1965 | Kampala, Uganda | >10 months but bigger study(1962-1965) | Benzathine penicilin+CQ PL(water) | Water | Protected=73, unprotected=84 CTRL=66, PROP=60 | N/A | Mean Hb higher in treated participants than control | N/A | | N/A |
| Eke et al., 2003 | Port Harcourt, Nigeria | 9months | PM (0.5 mg/kg·wk), PG (1.5 mg/kg·d), or PL (Vitamin C, 7 mg/kg·d) | Vit C | PM=36, PG=32, PL=29 | N/A | PM=7.60  PG=7.2 PL=6.8 | N/A | | N/A |
| Nwokolo et al., 2001 | Multicentre, Nigeria | 6 months | MQ (weekly 125 or 250mg) PG(100 or 200mg) | PG (daily 100 or 200mg) | 113 participants MQ(56) PG (57) | MQ=7.77 PG=7.83 | MQ=7.93 PG =8.23 | MQ+9.2 PG=9.1 | MQ=8.2 PG=8.6 | N/A |
| Nakibuuka et al., 2009 | Kampala, Uganda | 5 months | SP or CQ ( weekly) | CQ | 242 children SP=120 Chloroquine=122 | SP=7.3+ CQ=7.2 | N/A | N/A | | N/A |
